# Aluminosilicate Nanocomposites from Incinerated Chinese Holy Joss Fly Ash: A Potential Nanocarrier for Drug Cargos

**DOI:** 10.1038/s41598-020-60208-x

**Published:** 2020-02-25

**Authors:** Santheraleka Ramanathan, Subash C. B. Gopinath, M. K. Md Arshad, Prabakaran Poopalan, Periasamy Anbu, Thangavel Lakshmipriya

**Affiliations:** 1 Institute of Nano Electronic Engineering, Universiti Malaysia Perlis, 01000 Kangar, Perlis Malaysia; 2 School of Bioprocess Engineering, Universiti Malaysia Perlis, 02600 Arau, Perlis Malaysia; 3 School of Microelectronic Engineering, Universiti Malaysia Perlis, Pauh Putra, 02600 Arau, Perlis Malaysia; 4 Department of Biological Engineering, College of Engineering, Inha University, Incheon, 402-751 Republic of Korea

**Keywords:** Biochemistry, Environmental sciences

## Abstract

An incredible amount of joss fly ash is produced from the burning of Chinese holy joss paper; thus, an excellent method of recycling joss fly ash waste to extract aluminosilicate nanocomposites is explored. The present research aims to introduce a novel method to recycle joss fly ash through a simple and straightforward experimental procedure involving acidic and alkaline treatments. The synthesized aluminosilicate nanocomposite was characterized to justify its structural and physiochemical characteristics. A morphological analysis was performed with field-emission transmission electron microscopy, and scanning electron microscopy revealed the size of the aluminosilicate nanocomposite to be ~25 nm, while also confirming a uniformly spherical-shaped nanostructure. The elemental composition was measured by energy dispersive spectroscopy and revealed the Si to Al ratio to be 13.24 to 7.96, showing the high purity of the extracted nanocomposite. The roughness and particle distribution were analyzed using atomic force microscopy and a zeta analysis. X-ray diffraction patterns showed a synthesis of faceted and cubic aluminosilicate crystals in the nanocomposites. The presence of silica and aluminum was further proven by X-ray photoelectron spectroscopy, and the functional groups were recognized through Fourier transform infrared spectroscopy. The thermal capacity of the nanocomposite was examined by a thermogravimetric analysis. In addition, the research suggested the promising application of aluminosilicate nanocomposites as drug carriers. The above was justified by an enzyme-linked apta-sorbent assay, which claimed that the limit of the aptasensing aluminosilicate-conjugated ampicillin was two-fold higher than that in the absence of the nanocomposite. The drug delivery property was further justified through an antibacterial analysis against *Escherichia coli* (gram-negative) and *Bacillus subtilis* (gram-positive).

## Introduction

An increasing amount of fly ash is being produced at an alarming rate throughout the world, and it is not a simple subject to be neglected. More than 90 million tons of fly ash are being produced annually in India, China and the USA and ~50,000 acres of land are used for ash ponds^1^. Apart from coal combustion in thermal power plants, joss paper fly ash also shows a significant contribution toward tremendous fly ash accumulation. Joss paper has been used since the time of the six dynasties, and it is widely burned by the Chinese population during their festivals and funerals as a notable tradition. An estimated 90,000 to 220,000 tons of joss paper is burned each year. Although the exact figure has not been identified, it is one of the main causes of air pollution, predominantly in China and Taiwan. Efforts toward the invention of ecofriendly burning furnaces with high-efficiency incinerators, to reduce the release of heavy metals from fly ash, are not disregarded but have never met the expected objective^2,3^. Several efforts have been shown for the disposal or recycling of joss fly ash. The consumption of fly ash in cement and brick manufacturing improves the properties of the output and provides a method for recycling. However, the fly ash only amounts to 15–25% of the total cementitious mass. A large proportion of fly ash is disposed of in landfills but the search for other possible applications is needed to maximize waste consumption. In this regard, the synthesis of nanosized composites is shown to be a favorable method for recycling joss fly ash.

Since fly ash is rich in oxide-derived inorganic compounds, mainly silica and alumina, it has been extensively explored for the synthesis of aluminosilicate nanocomposites since the 1980s^4^. This nanocomposite is made up of a rigid three-dimensional framework of silicon with aluminum atoms and has a porous crystal structure with a large surface area/volume ratio compared to those of other naturally occurring nanocrystals^5^. The most popular synthetic method for preparing aluminosilicate nanocomposites using fly ash is a hydrothermal method at high temperature (300 to 700 °C) under alkaline conditions^6^. A Fusion-hydrothermal synthesis of the nanocomposite has been established, where a caustic agent is allowed to react with ash to generate highly active aluminosiliceous compounds. With respect to the hydrothermal method, studies have been conducted to investigate the optimal temperature, reaction time, and molar amount of caustic agent that results in high-performance aluminosilicate from fly ash waste^7^.

With a large surface area and porous cavity, aluminosilicates possess a high dispersion stability and biocompatibility and a higher reactivity in an appropriate nanoscale size for drug delivery applications. Studies have reported that aluminosilicate nanocomposites are nonspecific immunostimulators, as they are able to imitate the behavior of antigens that control the immune system of humans and animals. The nanocomposite is rich in hydroxyl groups that can modify and conjugate drugs on the surface. Successful loading of therapeutic drugs, antibacterial agents, nucleic acids and antioxidants to aluminosilicates has been well documented in the literature^8,9^. Therefore, aluminosilicate functionalization with drugs is considered a novel and promising nanovehicle for selective drug-carrier and drug-delivery applications^10–12^.

The present research is conducted to demonstrate the synthesis of aluminosilicate nanocomposites by recycling the abundance of joss fly ash. Since the burning of joss paper generates a large amount of ash and contributes to air pollution, this is an ecofriendly study that synthesizes aluminosilicate nanocomposites and proposes an efficient low-cost method to decompose joss fly ash. We focus on showing the drug-carrier and drug-delivery potentials of aluminosilicates synthesized from joss paper. The morphological and structural characteristics were investigated to justify that the synthesized aluminosilicates from joss fly ash have a high Si/Al ratio.

## Experimental

A few hundred grams of joss fly ash were collected from a joss paper burning furnace located in a Chinese temple in a northern state of Malaysia. Sodium hydroxide, sulfuric acid, Tween-20, Tris-buffer, (3-aminopropyl)triethoxysilane (APTES), 1-ethyl-3-(3-dimethylaminopropyl)-carbodiimide (EDC), and N-hydroxysuccinimide (NHS) were procured from Sigma Aldrich (USA). Enzyme-linked immunosorbent assay (ELISA) plates and horseradish peroxidase (HRP)-conjugated streptavidin were purchased from Thermo Scientific (USA). An ELASA coating buffer (5×) was procured from Biolegend (Japan). Bovine serum albumin (BSA) and 3,3′,5,5′-tetramethylbenzidine (TMB) were procured from Promega (USA). Biotinylated ampicillin aptamer with an ssDNA sequence of 5′-CACGGCATGGTGGGCGTCGTG-3′ was synthesized from a local supplier (Apical Scientific, Malaysia). The *Escherichia coli* and *Bacillus subtilis* strains were obtained from the School of Bioprocess Engineering, University Malaysia Perlis. A sterile disc was purchased from HiMedia Laboratories Pvt. Ltd. (Mumbai, India).

### Preparation of joss fly ash

Joss fly ash was collected 18 hours after the burning of joss paper. The collected joss ash was sieved; thus, impurities and dirt found in the ash were discarded. The cleaned ash was kept at room temperature for experimental purposes.

### Acidic treatment of joss fly ash

A 400-ml solution of 10% sulfuric acid (H_2_SO_4_) solution was prepared. Then, 20 g of joss fly ash was mixed in the prepared H_2_SO_4_ acid solution. The above pretreatment with a mildly acidic solution was performed to remove carbon and other toxic materials from the ash^13–17^. The mixture was stirred for 2 hours at 45 °C on a heating plate. After 2 hours, the solution and ash residue were separated by centrifugation. The acid-leached ash solution was centrifuged at 6000 rpm for 10 minutes, and the solution was collected in a clean beaker. On the other hand, the acid-leached ash residue was washed with distilled water a few times until a pH of 7 was attained. The residue was then kept in a vacuum oven at 80 °C for drying; the obtained powder was labeled J3 and used for further experiments.

A 300-ml solution of 5 M NaOH was prepared. The acid-leached solution was titrated using NaOH solution while stirring until the pH was neutral (pH 7). The pH of the solution was continuously measured using a pH meter. The NaOH solution was added dropwise to ensure that a pH of 7 was attained. The titration point was determined by the formation of tiny gels in the solution. Once a pH of 7 was attained, the solution was stirred for 18 hours. Continuous stirring ensured that the particles formed were completely homogenized and also to avoid particle aggregation. After 18 hours, the stirrer was stopped, and the solution was left for a few minutes until the gel formed and settled to the bottom of the beaker. The gel solution was centrifuged at 6000 rpm for 10 minutes to separate the gel from the solution. The collected gel was washed with 70% ethanol 3 times, followed by washing with distilled water twice. The gel was kept in a vacuum oven at 80 °C for complete drying. The sample was labeled J2 and kept at 4 °C for characterization analyses.

### Alkaline treatment of acid leached ash residue

A 400-ml solution of 2.5 M NaOH was prepared. The J3 sample obtained from the acid-treated joss fly ash was added to the prepared NaOH solution. The mixture was stirred for 4 hours at 100 °C on a hot plate. Then, the solution and ash residue were separated by centrifugation. The alkaline-leached solution with the residue was centrifuged at 6000 rpm for 10 minutes, and the dark alkaline-leached solution was collected in a clean beaker. The alkaline-leached ash residue was washed with distilled water a few times and then kept in a vacuum oven at 80 °C for complete drying. The obtained powder was labeled as the J4 sample and kept at 4 °C for characterization analyses.

**Figure 1:**
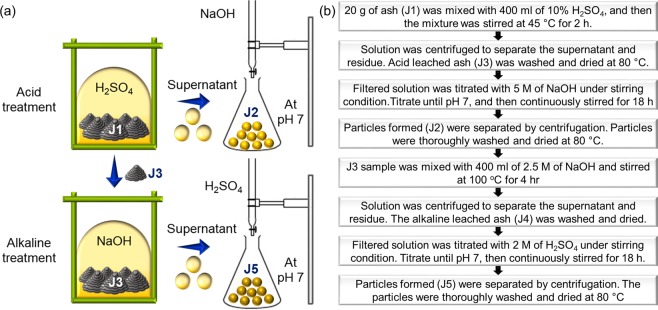
Experimental procedure conducted to synthesize aluminosilicate nanocomposites from joss fly ash. (**a**) Schematic illustration for synthesizing aluminosilicate nanocomposites (J5) from joss fly ash. (**b**) Experimental flow chart explains the performed synthesis process for the nanocomposite.

A 300-ml solution 2 M H_2_SO_4_ was prepared. The alkaline-leached solution was then titrated with the H_2_SO_4_ solution while stirring until the pH was neutral (pH 7). The titration point was determined by the formation of tiny gels in the solution, similar to the titration with the acid-leached solution. Once the solution attained a pH of 7, it was stirred for 18 hours. After that, the stirrer was stopped, and the solution was left for a few minutes until the gel formed and settled to the bottom of the beaker. The formed gel was washed and dried as stated in the above section. The sample was labeled J5 and kept at 4 °C for characterization analyses. Figure 1 illustrates the schematic for the experimental procedure performed in the research.

### Characterization of the aluminosilicate nanocomposite

Electron microscopy is one of the best and most widely used techniques to identify and characterize the particles formed from fly ash. Field emission transmission electron microscopy (FETEM) was used to inspect the size of the particles (JEM-2100F, JEOL, Japan). Joss fly ash and different experimental samples were diluted independently with 100 µL of ethanol, and a drop was dried on the sampling grid at room temperature. The dried samples were observed by FETEM to identify the morphology. Energy dispersive spectroscopy (EDX) was used to evaluate the elemental composition of the fly ash samples. FETEM analysis also permitted us to study the crystalline nature of the particles through selected area electron diffraction (SAED). In addition, the formation of aluminosilicates and their morphological characteristics were analyzed using field emission scanning electron microscopy (FESEM, Hitachi, S-4300 SE, Japan). The morphological images of each sample were analyzed with a high-energy electron beam at 15 kV and a working distance of ~4 mm. FESEM also provided an EDX analysis of the experimental samples. The surface roughness of the joss fly ash and its experimental samples were observed by atomic force microscopy (AFM) at a 5 µm magnification and 3000 Hz (NanoScope, Ica, Veeco, USA). 3D views of the particle surfaces were obtained to study the maximum height of the particles. A dynamic light scattering (DLS) analysis using a zeta potential and particle size analyzer was conducted to determine the stability and size distribution. Only the J5 sample, which was the extracted aluminosilicate, had a zeta potential analysis. The sample was dispersed in water to obtain a precise scattering intensity by the zeta analyzer (Photal Otsuka Electronics, ELC-Z model, Japan). The crystalline nature of aluminosilicate was examined by X-ray diffraction (XRD, DMAX-2500, Rigaku, Japan) using Cu K$\alpha$ (l = 1.54056 Å) as the radiation source. The sizes of the crystalline particles were analyzed by using different diffraction angles from 10–60° at 40 kV and 100 mA.). Fourier transform infrared (FTIR) spectroscopy was also conducted on the aluminosilicate to study its molecular configuration. The sample was prepared on a KBr pellet and scanned from 4000 to 650 cm^−1^ (Spectrum 65, Perkin Elmer, USA). Moreover, the experimental samples were analyzed by X-ray photoelectron spectroscopy (XPS) to identify the chemical bonding and elemental structure on the surface of the samples. XPS (Thermo Scientific, K-Alpha, UK) was equipped with an aluminum X-ray source operated at 72 W. Samples were prepared on a silicon (Si) wafer by dropping and drying a suspension. The thermal stability of the nanocomposite was analyzed by a thermogravimetry/differential thermal analysis (TG/DTA). Approximately 6 mg of aluminosilicate were heated from 20 to 600 °C, at a rate of at 10 °C/min^−1^, on an aluminum plate using a NETZSCH instrument (Leading Thermal Analysis, TG209, F3, Germany). Then, the weight loss was interpreted.

### Enzyme linked apta-sorbent assay (ELASA)

**Figure 2:**
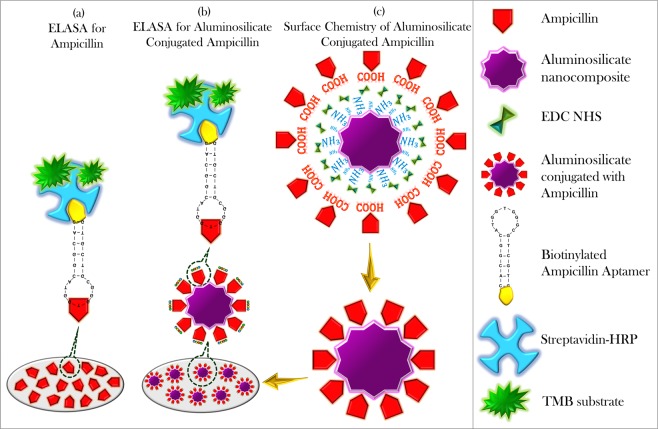
Schematic representation of ELASA conducted as a validation assay to investigate the ability of aluminosilicate nanocomposites synthesized from joss fly ash as a drug carrier. (**a**) ELASA for the detection of ampicillin. (**b**) ELASA for the detection of ampicillin-conjugated aluminosilicate nanocomposites as drug carriers. (**c**) Modification of aluminosilicate surface chemistry for ampicillin conjugation.

Ampicillin and the ampicillin captured on aluminosilicate were used as targets for an ELASA. Aluminosilicate was conjugated with ampicillin using (3-aminopropyl)triethoxysilane (APTES) and a 1:1 ratio of NHS (50 mM) and EDC (200 mM). The aptamer selected for ampicillin detection was a biotinylated aptamer with an ssDNA sequence of 5′-CACGGCATGGTGGGCGTCGTG-3′^18,19^, purchased from Apical Scientific (Malaysia). Figure 2 shows the schematic representation of ELASA designed to investigate the drug carrier potential of aluminosilicate nanocomposites synthesized from joss fly ash. Ampicillin was diluted to 2.5 mg/ml, whereas aluminosilicate-conjugated ampicillin was diluted to 125 µg/ml using 1X coating buffer. The diluted targets were immobilized onto the ELASA plate and kept at 4 °C overnight. The next day, washing was performed using 1X Tris-buffered saline (TBS) before conducting the blocking step. Then, 250 µL of 2% BSA (prepared using TBS buffer) was added into each well to mask the remaining ELASA surfaces and incubated for 1 hour at room temperature (RT). Next, consecutive washings were performed using a TBS-Tween 20 (TBST) buffer. The 1:200 diluted biotinylated ampicillin aptamer was added into each well and incubated for 1 hour at RT to permit interaction between the target and aptamer. Then, HRP-conjugated streptavidin at a 1:1000 dilution was allowed to bind to the biotinylated aptamer for 1 hour at RT. Finally, a TMB substrate for HRP was added, and a color change on the ELASA surface was observed. Absorbance readings were measured using a UV-Nanodrop (DS-11/DS-11, Denovix, USA) at 405 nm, and the results were evaluated. A control set was used for a comparison. Fifty microliters of all the active molecules were added, and 300 µL of the buffer solution was added for washing. Consecutive washings at each interval step of ELASA were performed three times, with each taking 10 minutes.

### Analysis of the bacterial inhibition potential of aluminosilicate nanocomposites

*E. coli* and *B. subtilis* were chosen as the bacteria to be tested with aluminosilicate nanocomposites. Figures S1a,b show the strains of cultured *B. subtilis* and *E. coli* on a nutrient agar, respectively. A disc diffusion assay was applied to examine the bacterial inhibiting property. The assay was performed as discussed in our earlier research^20^. Approximately 100 µL of bacterial inoculum were thoroughly spread on a nutrient agar plate using a glass spreader. Before incubating the plates, three sterile discs were evenly placed on each agar plate. The test ingredient was added precisely at the center of the disc. The plates were incubated at 37 °C overnight, and the bacterial inhibiting property of the test ingredients was observed.

In the study of bacterial inhibition with aluminosilicate, ampicillin was chosen as the positive control and conjugated with the nanocomposite. Both aluminosilicate alone and its conjugation with ampicillin were studied. One hundred milligrams of synthesized aluminosilicate were washed with KOH and then with water. Next, the cells were incubated with 1 ml of 2% APTES solution for 1 hour. APTES generates the formation of amine groups on the nanocomposite surface. After incubation, the mixture was centrifuged to remove the supernatant and washed with ethanol and then with water. A 500-µL sample of ampicillin (1 mg/ml) was activated by EDC and NHS linkers; then it was added into the above mixture and incubated for 1 hour. The carboxylic end in ampicillin and the amine end on the APTES-modified aluminosilicate formed an amide bond, and EDC and NHS were used as stabilizers. After incubation, the mixture was centrifuged to remove the supernatant and washed with water. Finally, 500 µL of water were added and mixed thoroughly. The aluminosilicate-conjugated ampicillin solution was used for a bacterial inhibition analysis. Herein, three main active ingredients were desired for bacterial inhibition: ampicillin as a positive control, aluminosilicate with an amine group, and aluminosilicate-conjugated ampicillin. Water was chosen as a negative control. Volumes at 5 µL, 10 µL and 20 µL of each active ingredient were added to each sterile disc, which were then placed on an agar plate spread with bacteria. The plates were incubated overnight at 37 °C to analyze the drug delivery properties of aluminosilicate nanocomposites synthesized from joss fly ash.

## Result and Discussion

### Aluminosilicate nanocomposite from joss fly ash

**Figure 3:**
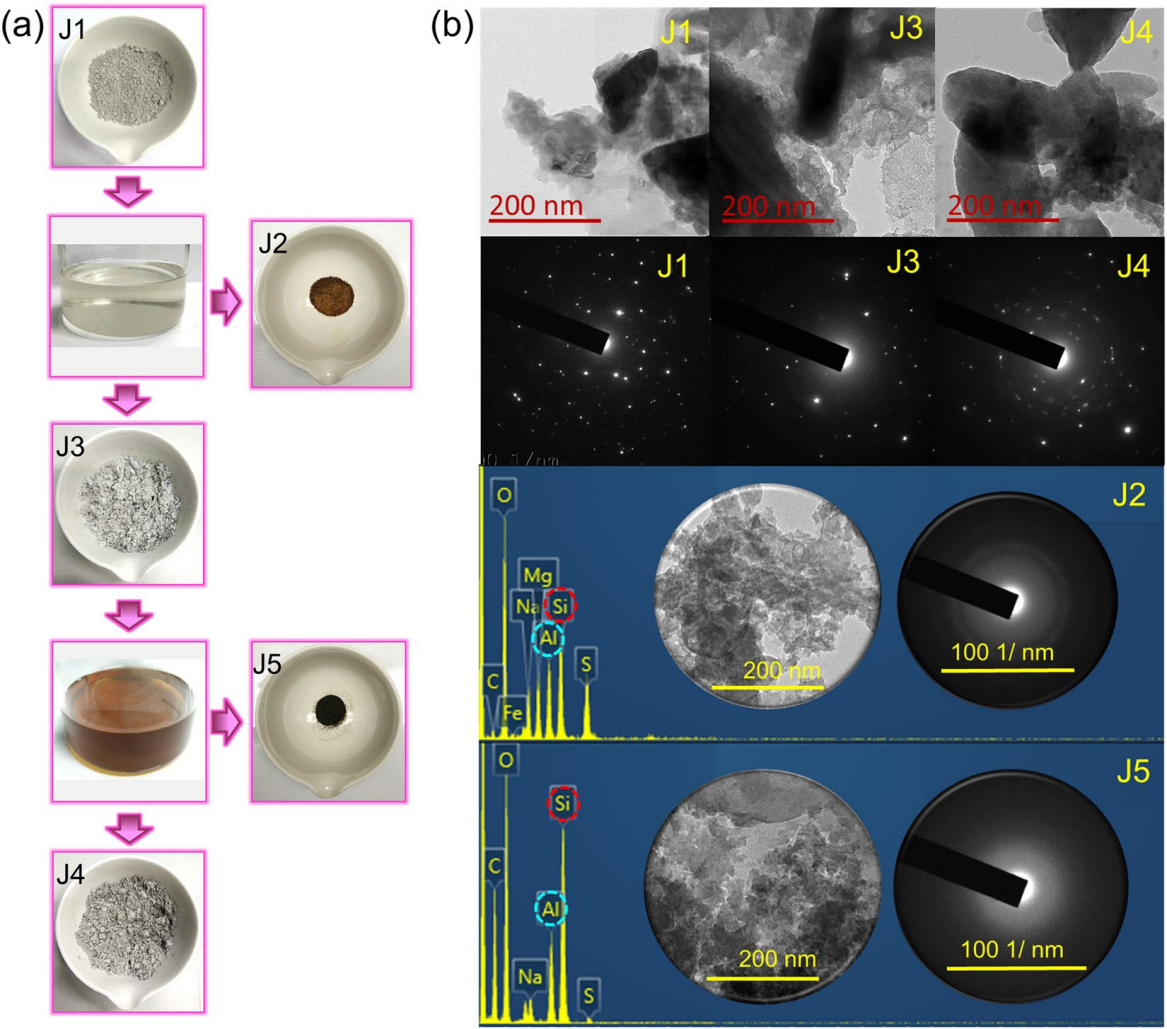
(**a**) Joss fly ash samples in the extraction of the aluminosilicate nanocomposite. J1 refers to the incinerated joss fly ash; J2 refers to the particles formed from the titrated acid-leached joss fly ash solution; J3 refers to the ash that undergoes an acid treatment; J4 refers to the joss sample that undergoes an alkaline treatment; and J5 refers to the aluminosilicate nanocomposite extracted from joss fly ash. (**b**) FETEM analysis of the joss fly ash samples. The analyzed FETEM images of J1, J3 and J4 at a 200 nm magnification are shown. Below the FETEM images, the SAED images are shown for the J1, J3 and J4 samples with respect to their FETEM images. FETEM analyses of the J2 and J5 samples are shown with EDX profiles, and the figure inset refers to the FETEM and SAED images.

In this study, joss paper ash [Fig. 3a (J1)] was first treated with mild sulfuric acid to remove excessive impurities. To obtain highly pure crystal-sized aluminosilicates, an acid treatment is an effective step before alkaline treatment. Figure 3a (J2) shows the particles extracted from the formed gel at pH 7. The acid-washed joss fly ash [Fig. 3a (J3)] was utilized for synthesizing the aluminosilicate nanocomposites. A 100 °C reaction temperature was investigated for the synthesis of aluminosilicate in an alkaline medium. Aluminosilicate nanocomposites have been theoretically explained as being synthesized by the dissolution of a gel in a basic medium. The joss fly ash sample collected from the alkaline treatment of the J3 sample was named J4 [Fig. 3a (J4)]. The formation of crystallized aluminosilicate nanocomposites [Fig. 3a (J5)] was clearly observed during the titration of alkaline-treated joss fly ash solution to neutral conditions. The solution was then washed thoroughly with ethanol and distilled water to remove precipitated sulfate salts and consequently to obtain pure nanocrystallized aluminosilicates^21–23^.

### Field emission transmission electron microscopy (FETEM)

FETEM analysis was conducted on the experimental samples of joss fly ash to study the morphology of each sample and obtain a precise size of the particle. Figure 3b shows the morphological structures observed under FETEM for the samples obtained during the synthesis of aluminosilicate from joss fly ash. J1, J3, and J4 show similarity to the morphologies of large colloidal particles, and a large range of particle sizes were observed. Among them, the FETEM image of the J3 sample shows a wave of aluminosilicate particle formation when the sample was obtained from the acid leached solution after a pH adjustment. The crystallinity of the particles formed in the J1, J3 and J4 samples was confirmed by selected area electron diffraction (SAED) images. Although the J1, J3 and J4 samples were represented as ash and its residues, the diffracted ring patterns observed in the SAED images indicate that the particles had a crystalline nature^24,25^. Next, FETEM of the J2 sample showed the formation of spherical-shaped particles that were closely attached to one another with a uniform size distribution. The SAED image of the J2 sample implies that the crystalline particles have a uniform distribution because a clear zone with no bright spots is visible^26^. In contrast, the FETEM image of the J5 sample shows the formation of small-sized and spherical-shaped particles, which are uniformly dispersed without much space between the particles. Similar to the J2 sample, a clear zone with no bright spots was observed in the J5 SAED image, which justified the crystalline property of the nanocomposite^20,27^. Uniformly dispersed aluminosilicate nanocomposites were shown to be attached to each other. The size of the aluminosilicate obtained from the FETEM image was ~25 nm. The synthesis of aluminosilicate was further verified by the EDX data of the J5 sample, which showed a higher elemental composition ratio of Si to Al compared to the Si to Al ratio in the J2 sample.

### Field emission scanning electron microscopy (FESEM)

**Figure 4:**
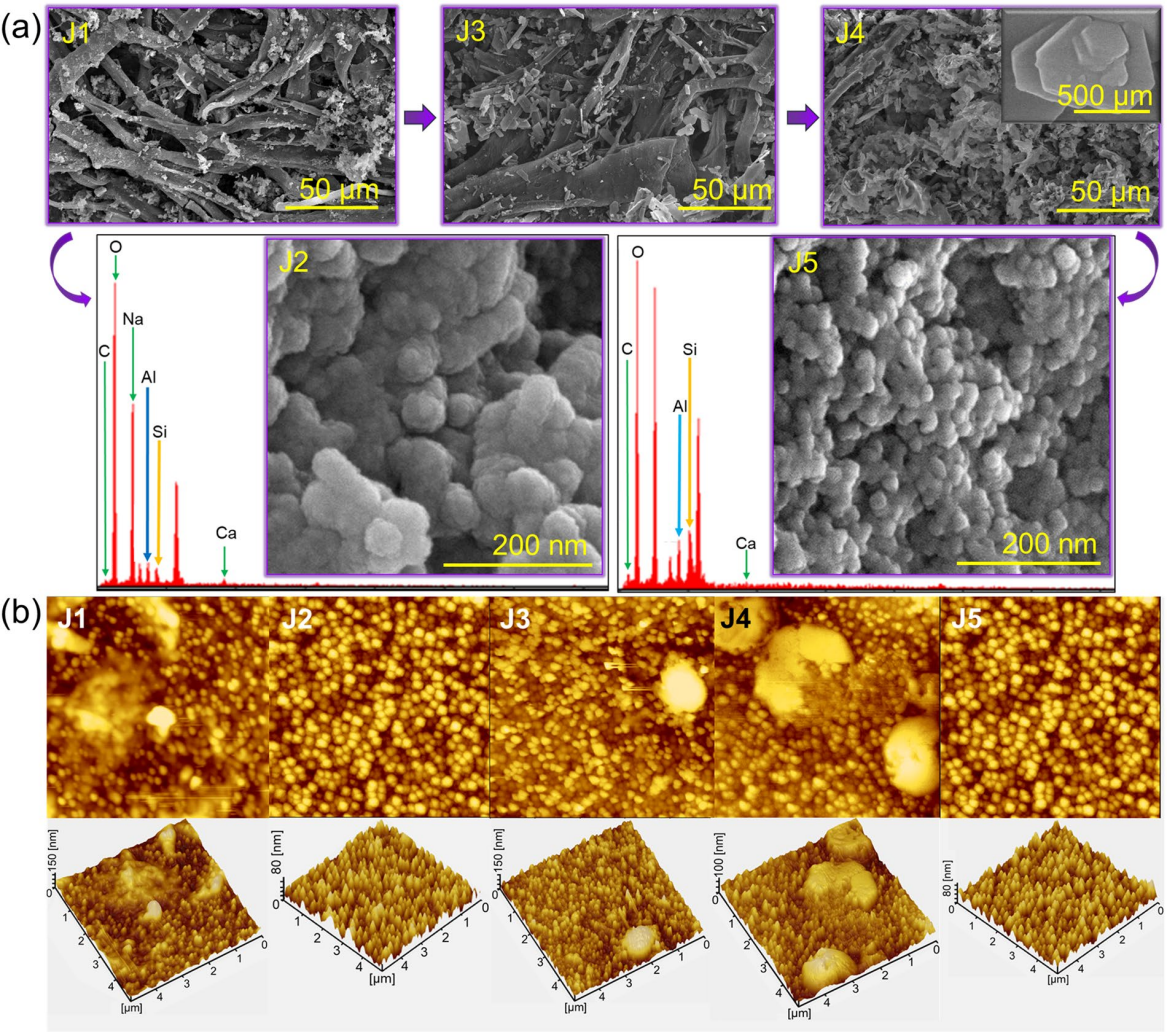
(**a**) FESEM analyses of the joss fly ash samples. J1, J3, and J4 sample FESEM images were examined at a 50 µm magnification. The figure inset in J4 refers to the magnified image of the J4 sample at 500 µm. EDX data for the J2 and J5 samples are shown, and the inset shows the FESEM images. (**b**) AFM analyses of the joss fly ash experimental samples in the synthesis of the aluminosilicate nanocomposites. AFM images of each sample are shown with the top view (top image) and 3D view (bottom image).

FESEM analysis revealed the shape of the particles with each sample formed during the synthesis of the aluminosilicate nanocomposite. Figure 4a (J1) shows the morphology of the incinerated joss fly ash without any further chemical modification. The image reveals agglomerations of hollow cage-structured particles with the presence of uneven tiny particles. The presence of tiny particles may be categorized as the impurities present in joss fly ash or the unburned ashes. Figure 4a (J2) reveals that spherical-shaped particles are agglomerated in large clusters. Since J2 sample particles formed after the fly ash was treated with a mild acid and titrated to reach a neutral pH, they were expected to contain solid particles of fly ash in the presence of a small amount of impurities. The particles were confirmed to have a large spherical-shape with an uneven distribution. Figure 4a (J3) shows an image of the acid-washed joss fly ash residue. Figure 4a (J4) shows the image of an alkaline-leached ash residue. The image shows a fine residue of joss fly ash, with no clearly shaped particles; instead, flat pentagon-shaped residues were observed. This proved that no appealing particles were found in an alkaline-leached residue. Figure 4a (J5) shows the precise spherical-shaped particles with a uniform distribution and no agglomeration. The particles with spherical shapes confirm the formation of aluminosilicate nanocomposites in the J5 samples with a size of ~25 nm. The result was further proven by EDX analysis. The predominant elements found in the J5 sample were Si and Al in a 13.24 to 7.96 ratio. Low quantities of carbon, oxygen and calcium were also observed. The EDX data obtained for the J5 sample by FESEM were essentially in good agreement with the EDX data obtained from FETEM in terms of the elemental composition and the Si to Al ratio, which proved the formation of crystalline aluminosilicate nanocomposites from joss fly ash^28^. Table 1 shows the EDX data of the experimental samples analyzed with FETEM and FESEM.

**Table 1:** EDX data joss fly ash samples from FESEM and FETEM.

Elements	Weighted Percentage (%)									
	FESEM					FETEM				
	J1	J2	J3	J4	J5	J1	J2	J3	J4	J5
Si	1.65	3.76	1.92	1.64	13.24	0.23	11.53	23.02	0.95	12.53
Al	—	4.76	0.55	0.45	7.96	0.54	8.64	10.72	0.16	5.30
C	31.95	2.38	5.36	9.48	6.17	32.08	6.35	14.72	20.42	37.71
O	23.75	41.75	48.73	39.97	71.43	40.41	51.60	50.32	51.43	42.49
Ca	42.65	2.30	43.43	48.45	1.20	26.03	—	0.64	21.36	—
Na	—	31.15	—	—	—	—	6.11	—	1.90	1.57
Mg	—	4.90	—	—	—	0.47	7.04	—	—	—
S	—	—	—	—	—	—	—	0.57	3.78	0.41

### Atomic force microscopy (AFM)

AFM analysis was performed for all the experimental samples to determine the surface roughness and the estimated particle size. The images of J1, J3 and J4 in Fig. 4b show that the particles in these samples are unevenly distributed and appear to be clustered with a large range in particle size. Based on the analysis, the surface roughness values of the J1, J3 and J4 samples were found to be high. This result might be due to the presence of impurities and dirt particles in the joss fly ash samples. However, AFM images of both the J2 and J5 samples appeared to be similar in terms of surface roughness, as the particles observed have sharp and rough edges with a uniform dispersion. The maximum height of the aluminosilicate nanocomposite in Fig. 4b (J5) was $\sim$ 80 nm with sharp and rough edges.

### Zeta analyzer

**Figure 5:**
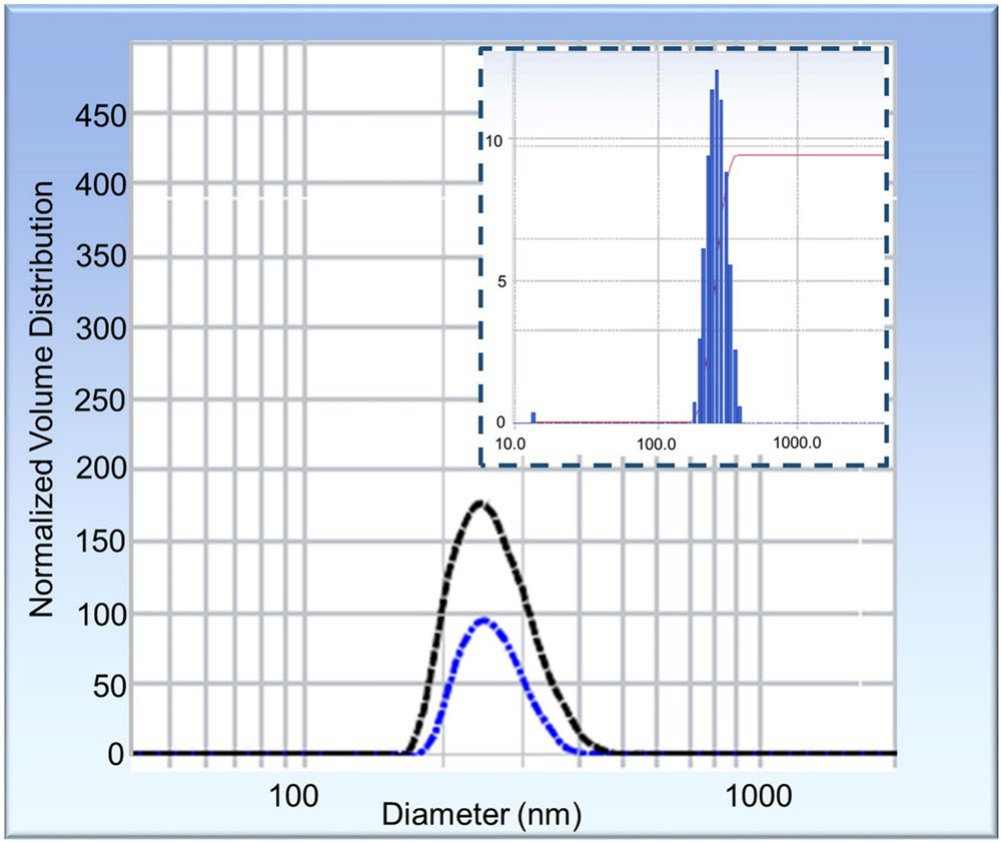
Size distribution of the aluminosilicate nanocomposite with a zeta analyzer. The duplicate analysis of aluminosilicate shows two curves in the normal distribution graph. The figure inset shows the zeta potential measurement of the aluminosilicate nanocomposite, indicating the stability of the nanocomposite in a suspension.

A zeta potential analyzer was used to examine the stability of a suspension of aluminosilicate nanocomposites synthesized from joss fly ash, and the size distribution was obtained based on dynamic light scattering (DLS). The value of the zeta potential measurement indicates the stability of a colloidal substance in suspension; a high measurement value represents high particle stability. From Fig. 5, the obtained zeta potential measurement obtained was +9.0 mV, which indicated that the synthesized aluminosilicate was highly stable in suspension. The stability in suspension is an important parameter for application in the biomedical and pharmaceutical industries, especially for the absorption and reabsorption of medicinal elements^29^. Moreover, the aluminosilicate size identified under DSL was 284 nm with a 0.317 polydispersity index (PDI). The principle of the zeta measurement is substantive because it measures a proportion of the analyzed sample (relative particle amount as a percentage where the total amount of particles is 100%). Thus, the large amount of particles measured through the zeta analysis justified the measurement of the particle proportion^29–33^. Unlike zeta potential measurements, a low PDI indicates a small and uniform size distribution of particles, whereas a high PDI value indicates a large and irregular distribution of particle sizes^34^. Hence, the low PDI value that was obtained confirmed the formation of small and uniformly sized aluminosilicates by recycling joss fly ash.

### X-ray diffraction spectroscopy (XRD)

**Figure 6:**
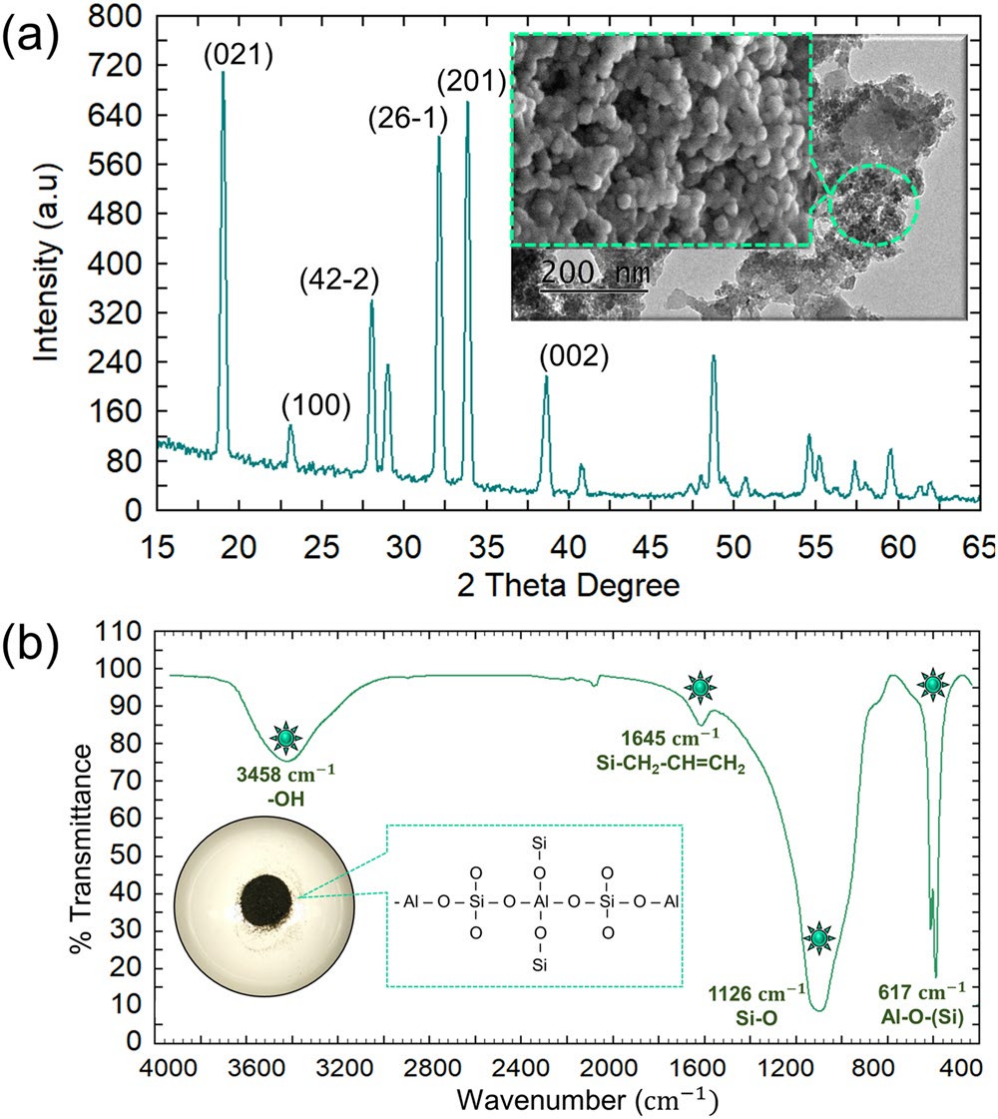
(**a**) XRD spectra of the aluminosilicate nanocomposite (J5 sample). The XRD spectra show three prominent peaks at 19.01, 33.81, and 32.13. (**b**) FTIR spectra of the aluminosilicate nanocomposite, which explains the chemical bonds examined in the nanocomposite.

Aluminosilicate nanocomposites synthesized from joss fly ash were examined with XRD to analyze the crystalline state based on the diffraction angles. Figure 6a shows the XRD spectra with three prominent peaks at 19.01°, 32.13° and 33.81°, corresponding to the (021), (26-1) and (201) diffraction lines, respectively. The observed diffraction angles were in good agreement with previous reports, thereby confirming the crystalline state of the aluminosilicates. As reported earlier, three notable diffraction peaks in the range of 15 to 35 degrees indicated the face-centered spherical crystalline index of the aluminoslicate^35,36^. However, the diffraction lines of (100) and (42-2) indicated lattice fringes that correspond to the planes of a cubic structure^37^. The crystallinity of the aluminosilicate may differ with changes in temperature^38^. Thus, the presence of faceted and cubic aluminosilicate crystals in the XRD spectra might be influenced by the temperature changes.

### Fourier transform infrared (FTIR) spectroscopy

The aluminosilicate synthesized from joss fly ash was characterized with FTIR spectroscopy to determine the functional groups in the molecule. Figure 6b shows the FTIR spectrum analyzed for aluminosilicate, and four prominent peaks are observed. A broadened peak was observed in the low frequency region at 617 cm^−1^, corresponding to the vibrations of the Al-O-(Si) bond due to the mixed silicon-aluminum-oxygen skeleton in the aluminosilicate tetrahedral configuration^39^. According to the literature, the prominent peak observed at 1126 cm^−1^ was the strong vibration of the Si-O bond^40,41^. The peak generated at 1645 cm^−1^ might be attributed to the isomeric groups of Si-CH_2_-CH = CH_2_ in the cycloalkane tetrahedral structure of aluminosilicate^42^. The broadening of the peak observed at 3458 cm^−1^ referred to the hydroxyl groups attributed to the vibration of water molecules^43–45^. Significant vibrations of Si-O and Al-O-(Si) functional groups were observed, indicating that the tetrahedral structure primarily appeared in the aluminosilicate nanocomposite.

### X-ray photoelectron spectroscopy (XPS)

**Figure 7:**
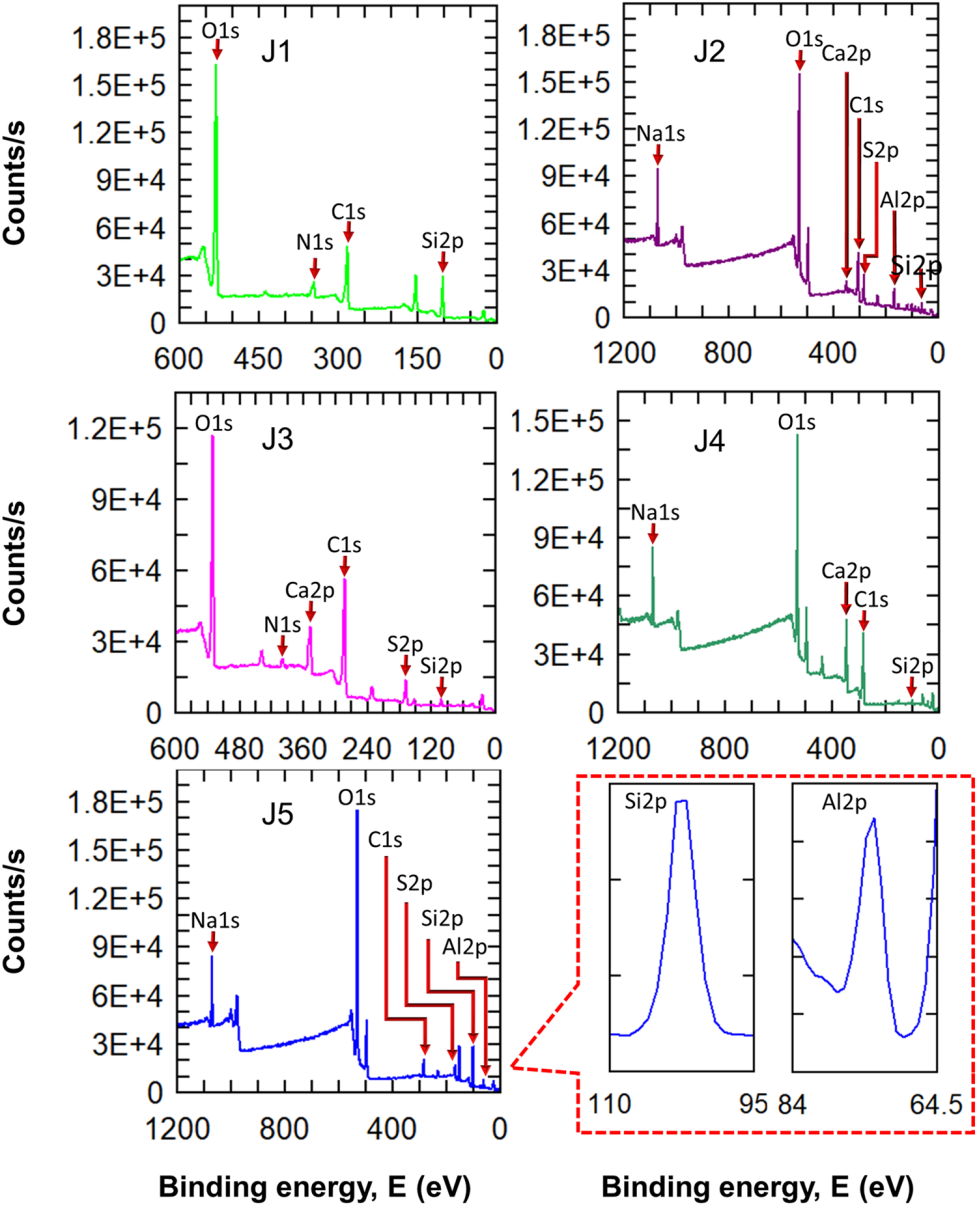
XPS spectra of the joss fly ash experimental samples. The analysis reveals the elemental composition found on the particle surface. Silicon and aluminum peaks in the XPS spectra are enlarged to emphasize the presence of silicon and aluminum in the aluminosilicate nanocomposite (J5).

The experimental samples of joss fly ash were examined with XPS to study the surface composition of the molecules formed in each step of the process for synthesizing aluminosilicate. The XPS spectra of J1 in Fig. 7 show the presence of Si 2p, representing the naturally appearing silicon on the surface of joss fly ash. After the joss fly ash undergoes an acid treatment, the XPS spectra of the J2 sample show variable compositions on the surface. It should be emphasized here that the Al 2p peak was higher than the Si 2p peak. The above verified that the ratio of aluminum to silicon was higher and was in good agreement with the similar ratio obtained from the EDX data in the FESEM analysis. The XPS spectra of the J3 and J4 samples were similar in their surface composition, the binding energies generated for the Si 2p peak were similar, at 102.03 and 101.53 count/s, respectively, and the binding energies of O 1 s, C 1 s, and Ca 2p were apparently visible. The Si on the surface of J3 was low compared to other samples, as it had not undergone alkaline treatment for aluminosilicate synthesis. Moreover, the low composition of Si on the J4 sample resembles the left-over impurities of the fly ash. The Si 2p composition in the J5 XPS spectra is higher than that in the other samples, with 3837.9 counts/s representing a greater composition of silicon on the particles. The O 1 s peak is the most significant in the XPS spectra of each sample analyzed, specifying that the joss fly ash particles are easily oxidized. The aluminosilicate with Si to Al atoms was connected by a tetrahedral framework of oxygen atoms; the above was justified by the prominent O 1 s peak that appeared in every sample, in particular, with the J5 sample, where a high-purity aluminosilicate nanocomposite was present. The rigid tetrahedral framework of Si-O in aluminosilicate may be responsible for the absence of Al in a greater amount than silicon on the surface.

### Thermogravimetry Analysis (TGA)

**Figure 8:**
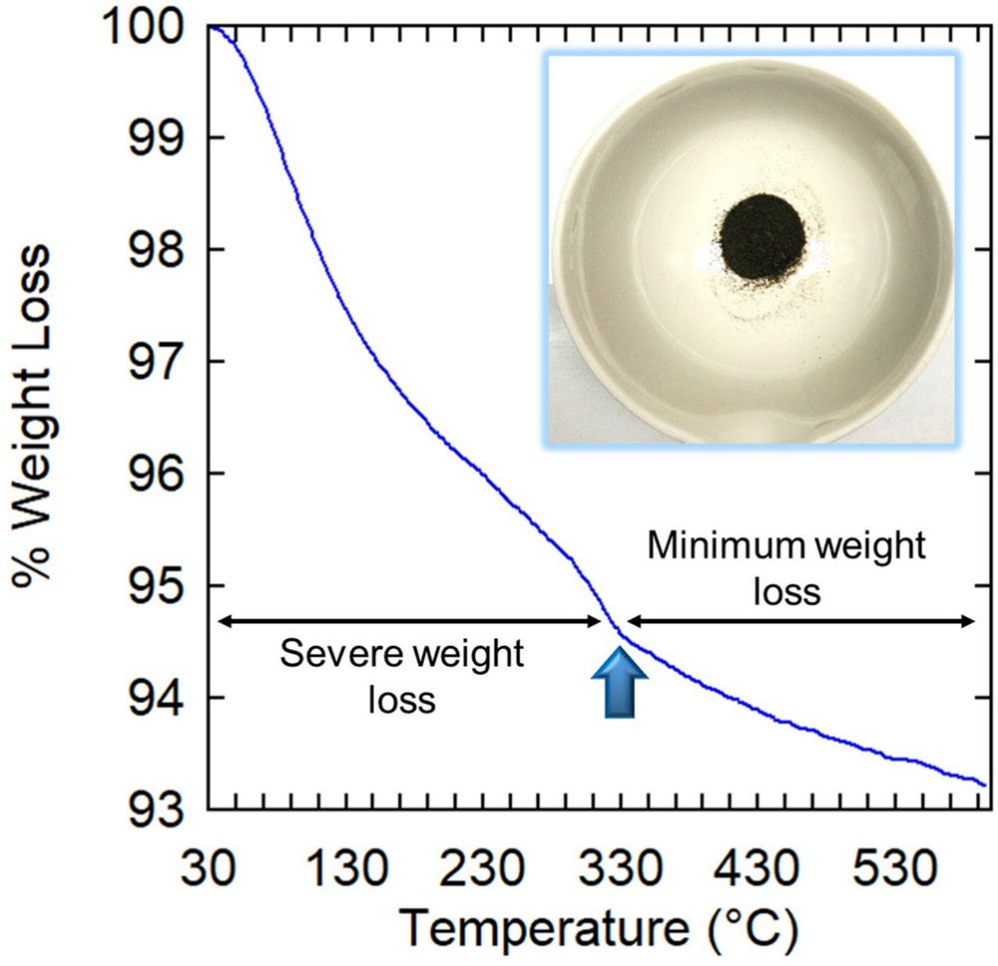
Thermal analysis of aluminosilicate nanocomposite. The TGA curve indicates the reduction of sample weight as the temperature increased from 30 °C to 600 °C.

The thermal stability of the aluminosilicate nanocomposite synthesized from joss fly ash was analyzed by thermogravimetry analysis. From Fig. 8, the linear weight loss of the aluminosilicate nanocomposite observed from 100 to 300 °C was due to the evaporation of water molecules from the nanocomposite, which was physically absorbed with the particles during the extraction process. From 300 to 600 °C, the weight loss of aluminosilicate was up to 1.8%. The ratio of Si to Al in aluminosilicate is directly proportional to its thermal stability. The minimal weight loss above 300 °C confirmed that aluminosilicates synthesized from fly ash had a high thermal stability, which was in agreement with other studies by Mourhly^43^. This enabled the particles to retain their structural configuration at high temperatures, which consequently proved that the extracted aluminosilicate nanocomposite from joss fly ash could be applicable in high-temperature processing.

### Enzyme linked apta-sorbent assay (ELASA)

**Figure 9:**
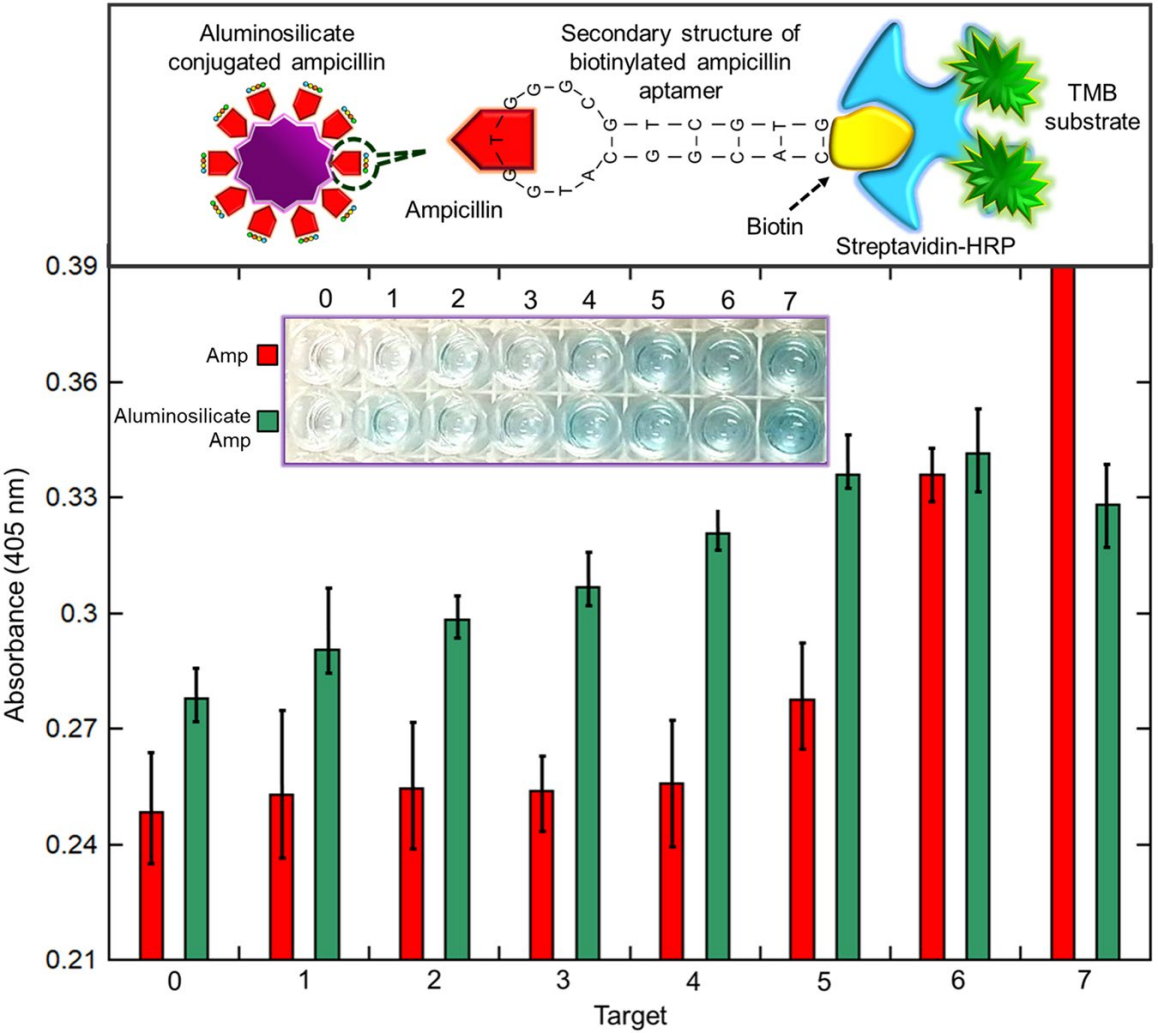
Absorbance readings of the ELASA assay. The ELASA assay was conducted on ampicillin- and aluminosilicate nanocomposite-conjugated ampicillin to identify the detection limit. For ampicillin, 1, 2, 3, 4, 5, 6 and 7 refer to 0.04, 0.08, 0.157, 0.313, 0.625, 1.25, and 25 mg/ml, respectively. For aluminosilicate-conjugated ampicillin, 1, 2, 3, 4, 5, 6 and 7 refer to 1.95, 3.91, 7.82, 15.63, 31.25, 62.5, and 125 mg/ml, respectively. Zero (0) indicates that the control was conducted with no target.

ELASA was carried out to determine the drug-carrier potential of the synthesized aluminosilicate nanocomposite. Aluminosilicate-conjugated ampicillin was examined to determine the limit of detection in comparison with only ampicillin by an ELASA assay. To recognize the occurrence of ampicillin, a biotinylated anti-ampicillin aptamer was used, followed by interaction with streptavidin-HRP conjugates. Figure 9 reveals the apparent detection of ampicillin at 0.625 mg/ml with 0.28 (optical density; O.D.) as the absorbance. Beyond that, the absorbance of ampicillin noticeably increased from 0.28 to 0.33 and to 0.43 at 2.5 mg/ml. In agreement with the absorbance, an obvious color change was observed at 0.625 mg/ml on the ELASA plate, and the intensity of the blue color increased up to the highest concentration of ampicillin. The ELASA results of ampicillin emphasize that the lowest limit of detection by its aptamer was 0.625 mg/ml. In contrast, a constant elevation in the absorbance reading was notable for the aluminosilicate-conjugated ampicillin. It specified that the aptamer was able to detect the target at a low concentration, as a light blue color was observed at 1.95 µg/ml. Then, the intensity of blue was observed to be increased at 15.63 µg/ml, which perfectly corresponded to the rise in absorbance from 0.30 to 0.32, for concentrations from 7.82 to 15.63 µg/ml. As such, the lowest detection limit of aluminosilicate-conjugated ampicillin was at 1.95 µg/ml, which was lower than the detection limit with only ampicillin. The comparative detection limits of ampicillin and the conjugated aluminosilicate explained that the nanocomposite further upgraded the detection of the target by the aptamer, which might be supported by the enhanced catalytic and ion-exchange processes. At the lowest concentration of both analytes, the optical density of aluminosilicate-conjugated ampicillin was two-fold higher than that of ampicillin, indicating that the detection limit of aluminosilicate with ampicillin was higher. Aluminosilicate enabled a large number of ampicillin molecules to be broadly exposed on its surface due to its large surface area/volume. This enabled the aptamer to easily bind to ampicillin at the lowest concentration. At an aluminosilicate-conjugated ampicillin concentration of 125 µg/ml, the aptamers bound to all the available target molecules caused the molecules to become saturated. Finally, the ELASA assay confirmed that the attachment of aluminosilicate with ampicillin had the ability to detect small biomolecules, complex targets and cells, suggesting potential applications in the biomedical and pharmaceutical industries^21,46–48^.

### Joss paper-extracted aluminosilicate nanocomposites for use in drug-delivery

The potential of aluminosilicate synthesized from joss fly ash in drug delivery requires the capacity of the nanocomposite to conjugate with drugs and the ability to load and release the drugs at the desired location. In the present research, the above requirements were investigated through bacterial inhibition. Ampicillin is a commonly known antibiotic for treating pathogenic infections in humans and animals. Herein, ampicillin provided active ionizable functional groups, enabling easy conjugation with electrochemically charged aluminosilicate and permitting the nanocomposite to behave as a drug carrier^49^. The zone of inhibition shown by the aluminosilicate-conjugated with ampicillin resembled its potential in the drug-delivery process.

Figure S2a–d shows the cultured plates of *E. coli* and *B. subtilis* against ampicillin and aluminosilicate-conjugated ampicillin. There was no doubt that there was an obvious zone of bacterial inhibition shown by ampicillin against both *B. subtilis* [Figure S2a] and *E. coli* [Figure S2c], thus, it demonstrated excellent anti-pathogenic properties. Among *E. coli* and *B. subtilis*, ampicillin demonstrated better bacterial inhibition against *E. coli*. The inhibition zone observed with 5 µL of ampicillin on the *E. coli* cultured plate was 2 mm; however, zero bacterial inhibition was observed against *B. subtilis*. The differences might be due to the variation in the thickness of the peptidoglycan layer of the gram-negative (*E. coli*) and gram-positive (*B. subtilis*) bacteria. Since the peptidoglycan layer in *B. subtilis* was thicker, the antibiotic could not penetrate the bacterial cell wall as easily as it could with *E. coli* due to it having a thin peptidoglycan layer^20,23,27,48^. At the highest concentration of ampicillin (0.5 µg/µL), the zone of inhibition examined against *E. coli* was 6.5 mm, whereas the bacterial inhibition observed against *B. subtilis* was 2 mm, indicating that the bacterial inhibition of ampicillin against *E. coli* was 3-fold higher than the inhibition shown against *B. subtilis*.

Table 2 indicates that no zone of bacterial inhibition was observed on the cultured plates for *B. subtilis* [Figure S3a] and *E. coli* [Figure S3b] with only aluminosilicate at any concentration, indicating that the nanocomposite does not possess an antibacterial property^21,50,51^. Then, the antibacterial properties of the aluminosilicate-conjugated ampicillin against B. subtilis and E. coli were examined, as shown in Fig. S2b,d, respectively. The bacterial inhibition observed with 5 µL (2.5 µg) on *E. coli* was 1.75 mm, whereas on *B. subtilis*, it was 1 mm. It was apparent that the zone of bacterial inhibition shown by aluminosilicate-conjugated ampicillin against *E. coli* was larger than that against *B. subtilis*, illustrating the variation in the cell wall of the bacteria. Figure S4 shows the microscopic images of gram-positive and gram-negative bacteria and their mechanism in antimicrobial activities due to variations in the thicknesses of the peptidoglycan layer. With 10 µL (5 µg) of the analyte, the bacterial inhibition observed against *E. coli* was 3 mm, yet in *B. subtilis*, it was 1.25 mm, which was less than half of the value against *E. coli*. Similarly, with 20 µL (10 µg/µL), the bacterial inhibition shown against *B. subtilis* was 2.25 mm, but against *E. coli*, it was doubled to 4 mm. The results justified the above explanation that the bacterial inhibition against gram-positive *B. subtilis* was less than the bacterial inhibition against gram-negative *E. coli* with all concentrations of the aluminosilicate-conjugated ampicillin. The zone of inhibition shown by 10 µL ampicillin against *E. coli* was the same as the bacterial inhibition (4 mm) shown by 20 µL of the aluminosilicate with ampicillin. The results validate that the aluminosilicate synthesized from joss fly ash has great potential for delivering drugs, since it has a similar zone of bacterial inhibition at twice the concentration of ampicillin. Similarly, the strength of the 10 µL and 20 µL samples of aluminosilicate-conjugated ampicillin was similar to that of ampicillin in inhibiting the growth of *B. subtilis*. Although aluminosilicate-conjugated ampicillin did not show higher bacterial inhibition than ampicillin at equal concentrations, it enabled drug release in the presence of pathogenic bacteria. The zone of bacterial inhibition was close enough to ampicillin, which proved the excellent ion-exchange property,due to the large surface area of the aluminosilicate nanocomposite and enhanced drug delivery^52,53^. Therefore, aluminosilicate synthesized from joss fly ash, demonstrated high potential for drug delivery based on its ability to encapsulate and release drugs.

**Table 2:** Measurements on the zone of bacterial inhibition triggered by ampicillin, aluminosilicate modified with APTES and aluminosilicate conjugated with ampicillin. The values of bacterial inhibition were presented from triplicate experiments.

Analytes	*E. coli*			*B. subtilis*		
	5 µL	10 µL	20 µL	5 µL	10 µL	20 µL
**Zone of Inhibition, mm**						
Ampicillin	2 (±0.25)	4 (±0.5)	6.5 (±1.25)	0	1.5 (±0.5)	2.25 (±0.25)
Aluminosilicate Nanocomposite	0	0	0	0	0	0
Aluminosilicate Ampicillin Conjugation	1.75 (±0.25)	3 (±0.5)	4 (±0.25)	1 (±0.25)	1.25 (±0.1)	2 (±0.15)

## Conclusion

Highly pure aluminosilicate nanocomposites were synthesized from joss fly ash waste in an appropriate nanoscale size of ~25 nm that appeared to be uniformly distributed as sphere-shaped particles. The efficiency of synthesizing highly pure aluminosilicate from joss fly ash was proven through the ratio of silica to aluminum (13.21 to 7.96), which was obtained by an EDX analysis during FESEM. Analysis of aluminosilicates by AFM, XRD and zeta analysis proved the extraction of crystalline nanocomposites with sharp edges and a high stability in a suspension. FTIR and XPS analyses established the structural composition of elements on the surface, justifying the presence of silica and aluminum. A TGA analysis of the thermal stability of the aluminosilicate implied the capacity of the nanocomposite to resist high temperatures. An ELASA demonstrated a two-fold higher detection limit of aluminosilicate-conjugated ampicillin compared to that of ampicillin. Moreover, the antibacterial analysis of aluminosilicate-conjugated ampicillin demonstrated the excellent drug-delivery property of the extracted aluminosilicate. The analyses demonstrate that the aluminosilicate from joss fly ash has notable potential as a promising drug carrier and for drug delivery in the biomedical and pharmaceutical industries. The research emphasized the utilization of joss fly ash waste for the synthesis of highly pure aluminosilicate nanocomposites, which showed excellent performance as biocatalysts.

## Supplementary information

Supplementary Information.

## Data Availability

Relevant data are available in the Supplementary Source file.
