# Supplementary material for: Aluminosilicate Nanocomposites from Incinerated Chinese Holy Joss Fly Ash: A Potential Nanocarrier for Drug Cargos
Source: Sci Rep. 2020 Feb 25;10:3351. doi: 10.1038/s41598-020-60208-x (PMC7042258; doi:10.1038/s41598-020-60208-x)
Supplement: Supplementary file 1 — Supplementary Information. [file 41598_2020_60208_MOESM1_ESM.pdf]

Supplementary Information

Aluminosilicate Nanocomposite from Incinerated Chinese Holy Joss Fly Ash: A Potential Nanocarrier for Drug Cargos

Santheraleka Ramanathan[1], Subash C.B. Gopinath[1,2,*], M.K. Md Arshad[1,3], Prabakaran Poopalan[3], Periasamy Anbu[4], Thangavel Lakshmipriya[1]

[1]Institute of Nano Electronic Engineering, Universiti Malaysia Perlis, 01000 Kangar, Perlis, Malaysia.
[2]School of Bioprocess Engineering, Universiti Malaysia Perlis, 02600 Arau, Perlis, Malaysia.
[3]School of Microelectronic Engineering, Universiti Malaysia Perlis, Pauh Putra, 02600 Arau, Perlis, Malaysia.
[4]Department of Biological Engineering, College of Engineering, Inha University, Incheon 402-751, Republic of Korea.

Correspondance to:

Asso. Prof. Dr. Subash C.B. Gopinath
(subash@unimap.edu.my)

Figure S1

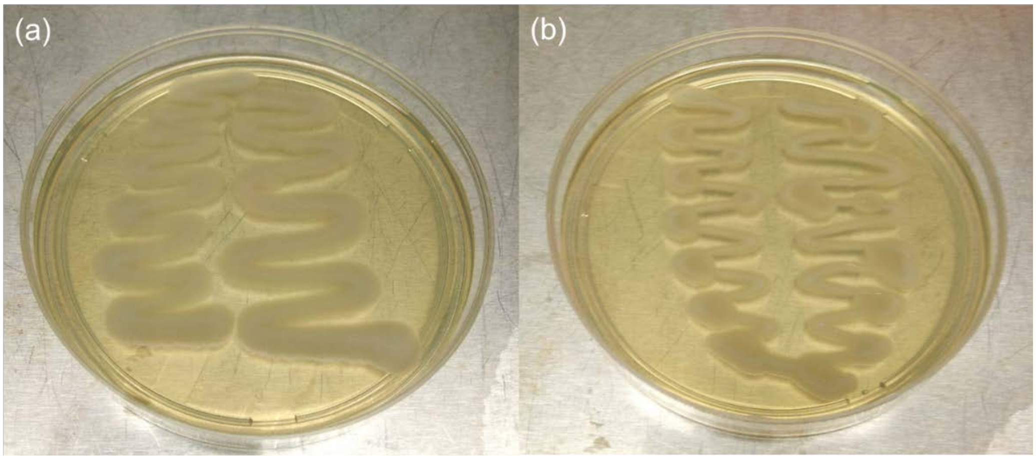

Figure S1: Cultured bacteria plates, (a) *B. subtilis* and (b) *E.coli* were used for the microbial inhibition analysis.

Figure S2

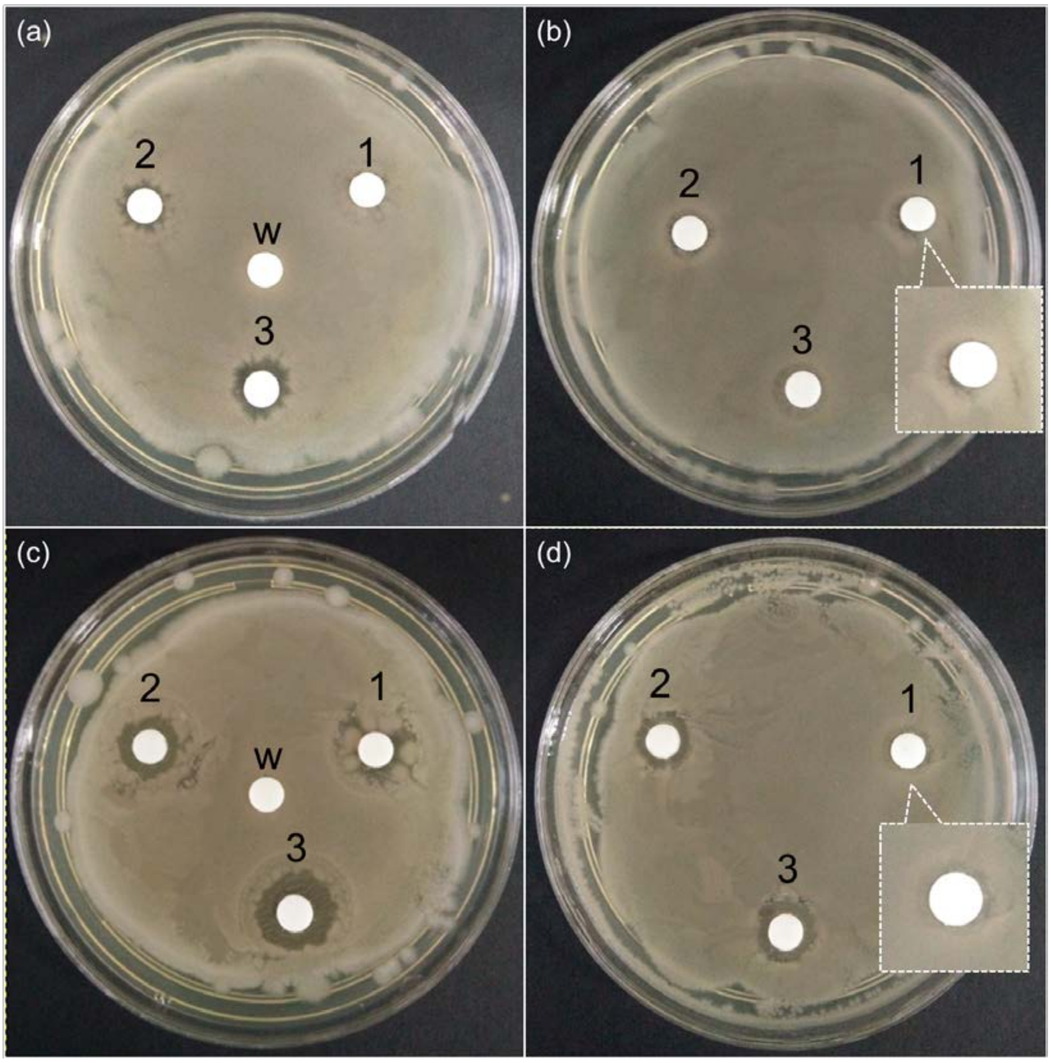

Figure S2: Cultured plates of *E. coli* and *B. subtilis* for antibacterial analysis. The analysis conducted for drug delivery examination of aluminosilicate nanocomposite conjugated with anti-pathogenic drug. (a) Ampicillin against *B. subtilis*; (b) aluminosilicate conjugated with ampicillin investigated for bacterial inhibition against *B. subtilis*; (c) Ampicillin against *E. coli* (d) aluminosilicate conjugated with ampicillin investigated for antibacterial property on *E. coli* culture agar plate. For ampicillin and aluminosilicate conjugated with ampicillin, 1, 2, and 3 on culture plates refer to 2.5 µg/5 µL, 5 µg/10 µL, and 10 µg/20 µL, respectively. The final volumes were kept uniform.

Figure S3

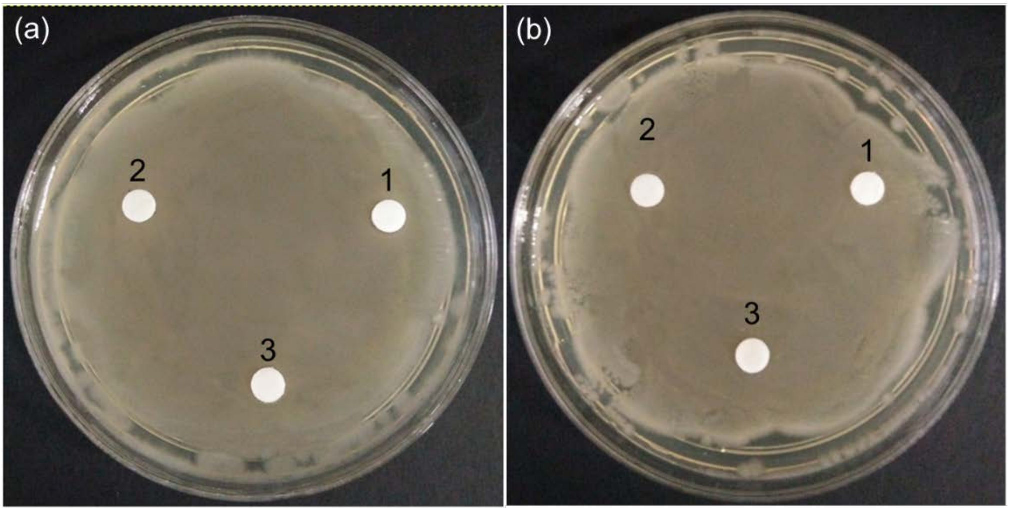

Figure S3: Aluminosilicate nanocomposite modified with APTES solution investigated for antimicrobial property against (a) *B. subtilis* and (b) *E. coli.* No microbial inhibition was observed indicates the inability of aluminosilicate in inhibiting pathogenic microorganisms. For aluminosilicate modified with APTES, 1, 2, and 3 on culture plates refer to 0.5 mg/5 µL, 1 mg/10 µL, and 2 mg/20 µL, respectively. The final volumes were kept uniform.

Figure S4

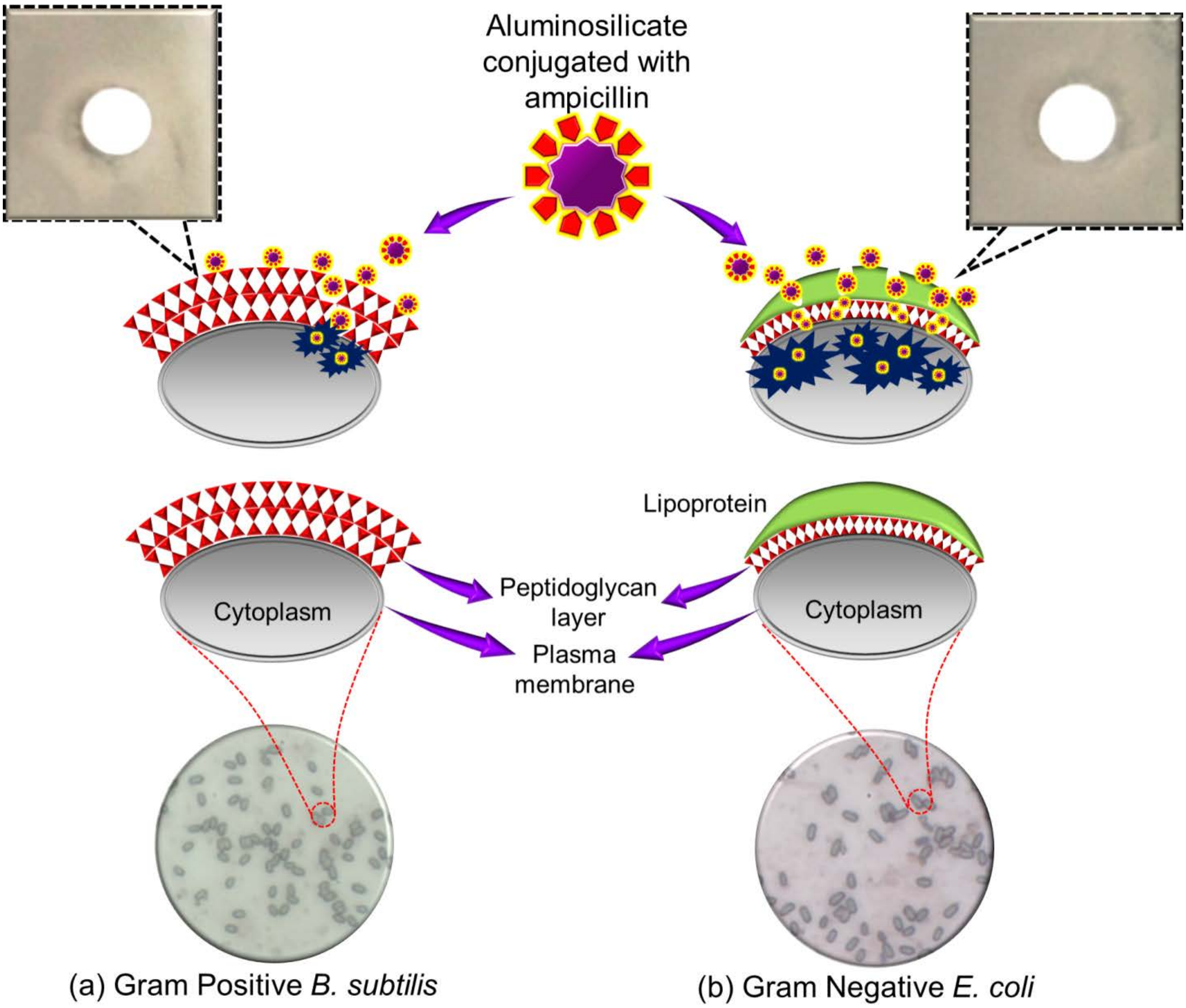

Figure S4: Differences between gram positive (*B. subtilis*) and gram negative (*E. coli*) on bacterial inhibition. (a) Gram stained image of *B. subtilis*. The purplish blue stain observed in the microscopic image justifies that *B. subtilis* is gram positive bacteria with thick peptidoglycan layer. (b) Gram stained image of *E. coli*. The red color stain in the image evidences that *E. coli* is gram negative bacteria with thin peptidoglycan layer. As illustrated in the image, *E. coli* with thin peptidoglycan layer in its cell wall allows facile penetration of ampicillin conjugated aluminosilicate and shows large zone of bacterial inhibition. *B. subtilis* with thick peptidoglycan layer strains the penetration of drug conjugated nanoparticle.
